# Supplementary material for: Spatial Autocorrelation Aware Resampling Improves Cell-Cell Interaction Inference in Spatial Transcriptomics Data
Source: bioRxiv. 2026 Jul 10:2026.07.06.736800. Preprint. [Version 1] doi: 10.64898/2026.07.06.736800 (PMC13370955; doi:10.64898/2026.07.06.736800)
Supplement: Supplement 3 [file NIHPP2026.07.06.736800v1-supplement-3.pdf]

bioRxiv preprint doi: https://doi.org/10.64898/2026.07.06.736800; this version posted July 10, 2026. The copyright holder for this preprint (which was not certified by peer review) is the author/funder, who has granted bioRxiv a license to display the preprint in perpetuity. It is made available under a CC-BY 4.0 International license.

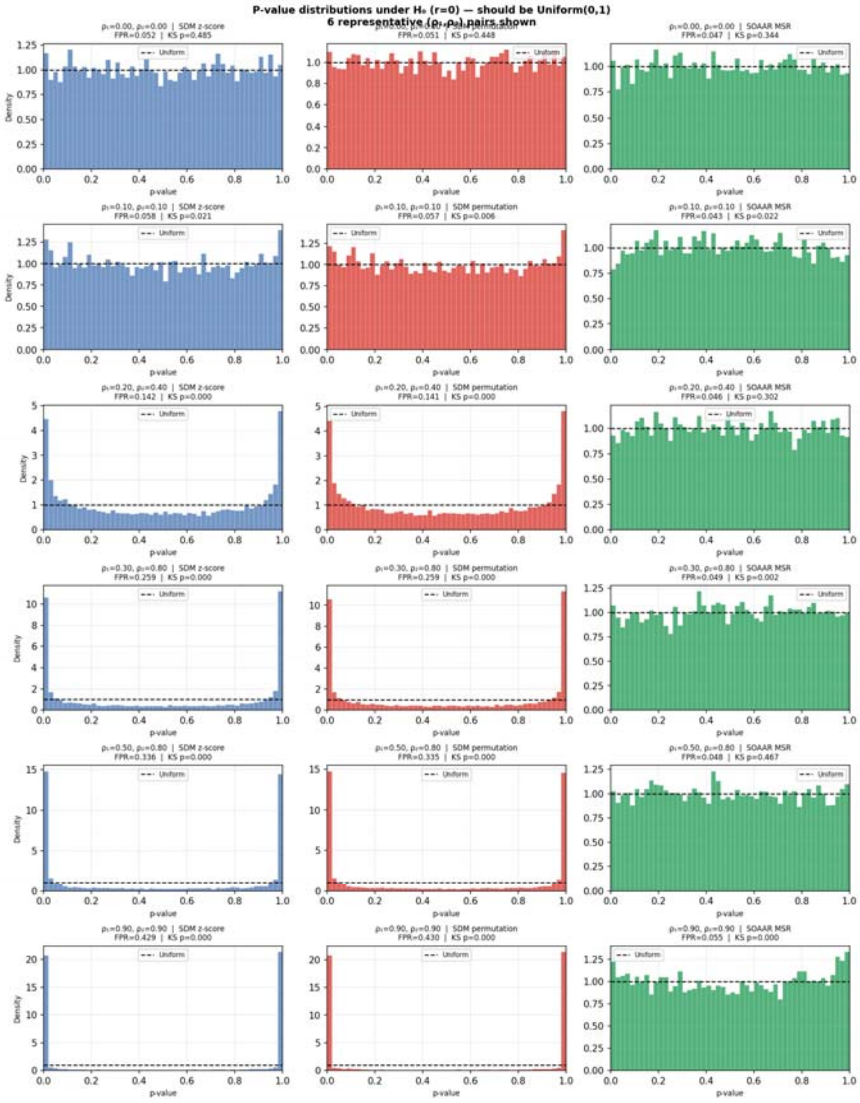

Figure S1 - P-value histograms for SpatialDM z-score (blue, left column), SpatialDM permutation (red, center column), and SOAAR MSR (green, right column) across six representative pairs of spatial autocorrelation coefficients (ρ_1, ρ_2), evaluated on 10,000 simulated null gene pairs per coefficient pair ($r = 0$) on a 20×20 rook grid.

A. At no autocorrelation ($\rho_1 = \rho_2 = 0$), all three methods produce approximately uniform p-value distributions with FPR near 0.05.
B. At mild autocorrelation ($\rho_1 = \rho_2 = 0.1$), SpatialDM's methods show a slight increase in FPR, while SOAAR remains well-calibrated.
C. At moderate autocorrelation ($\rho_1 = 0.2$, $\rho_2 = 0.4$), SpatialDM methods show clear false positive inflation, while SOAAR remains close to uniform.
D. At even higher autocorrelation ($\rho_1 = 0.3$, $\rho_2 = 0.8$), SpatialDM methods show severe false positive inflation, while SOAAR shows minor deviation from uniformity.
E. At ($\rho_1 = 0.5$, $\rho_2 = 0.8$), SpatialDM inflates false positives even further while SOAAR retains its calibration.
F. At the most extreme autocorrelation regime ($\rho_1 = \rho_2 = 0.9$), SpatialDM's method reach a false positive rate over 8 times that of the empirical false positive rate while SOAAR shows only a moderate degree of inflation.

bioRxiv preprint doi: https://doi.org/10.64898/2026.07.06.736800; this version posted July 10, 2026. The copyright holder for this preprint (which was not certified by peer review) is the author/funder, who has granted bioRxiv a license to display the preprint in perpetuity. It is made available under a CC-BY 4.0 International license.

Supplementary Table 1 – Combined results across all 10X Visium datasets for all tested ligand-receptor interactions with the following fields:

- Sample: The sample ID
- Response_group: Response status to immunotherapy
- Ligand: Ligand identity
- Receptor: Receptor identity
- Regime: Secreted signaling/contact signaling/ECM interaction label
- Concordant: Called significant by both SpatialDM and SOAAR
- sdm_only: Called significant by only SpatialDM
- soaar_only: Called significant by only SOAAR
- neither: Called significant by neither SpatialDM nor SOAAR
- soaar_sig: Called significant by SOAAR (soaar_fdr < 0.05)
- soaar_pval: uncorrected p-value based on SOAAR's test
- soaar_fdr: BH corrected p-value based on SOAAR's test
- z_score: z-score of SOAAR p-value based on null mean and variance calculated by SOAAR
- sdm_sig: Called significant by SpatialDM (sdm_fdr < 0.05)
- sdm_pval: uncorrected p-value based on SpatialDM's test
- sdm_fdr: BH corrected p-value based on SpatialDM's test
- observed: Observed bivariate Moran's I
- null_mean: Mean bivariate Moran's I of null distribution
- null_var_msr: Bivariate Moran's I variance based on SOAAR's MSR framework
- var_random_null: Bivariate Moran's I variance based on SpatialDM
- log10_var_ratio: log10 of the variances calculated by both methods
- pearson_lr: Pearson correlation between ligand and receptor

Supplementary Table 2 – Combined results across all CosMx Fields of View for all tested ligand-receptor interactions with immune cells as either senders or receivers with the following fields:

- Ligand: Ligand identity
- Receptor: Receptor identity
- Annotation: Secreted signaling/contact signaling/ECM interactions
- weight_used: Weight matrix used, W_secreted or W_contact
- I_obs: Observed bivariate Moran's I
- p_soaar: uncorrected p-value based on SOAAR's statistical test
- p_sdm_z: uncorrected p-value based on SpatialDM's analytic z-score calculation
- p_sdm_perm: uncorrected p-value based on SpatialDM's permutation
- immune_mode: Whether an immune cell was the sender or receiver
- fdr_soaar: BH corrected p-value based on SOAAR's statistical test
- fdr_sdm_z: BH corrected p-value based on SpatialDM's analytic z-score calculation
- fdr_sdm_perm: BH corrected p-value based on SpatialDM's permutation
- sig_soaar: Called significant by SOAAR's statistical test (fdr_soaar < 0.05)
- sig_sdm_z: Called significant by SpatialDM's analytic z-score (fdr_sdm_z < 0.05)
- sig_sdm_perm: Called significant by SpatialDM's permutation (fdr_sdm_perm < 0.05)
- run: Tumor microarray name and index
- fov: Field of view index

bioRxiv preprint doi: https://doi.org/10.64898/2026.07.06.736800; this version posted July 10, 2026. The copyright holder for this preprint (which was not certified by peer review) is the author/funder, who has granted bioRxiv a license to display the preprint in perpetuity. It is made available under a CC-BY 4.0 International license.

Supplementary Table 3 – Linking TMA+fov identities from CosMx assay to deidentified patients and their immunotherapy response status

- FOV_ID: run + fov from table 2 concatenated and underscore delimited
- Patient_ID: Deidentified patient, indicated by letter
- Response: Immmunotherapy response status
